# RNAi Reduces Expression and Intracellular Retention of Mutant Cartilage Oligomeric Matrix Protein

**DOI:** 10.1371/journal.pone.0010302

**Published:** 2010-04-22

**Authors:** Karen L. Posey, Peiman Liu, Huiqiu R. Wang, Alka C. Veerisetty, Joseph L. Alcorn, Jacqueline T. Hecht

**Affiliations:** 1 Department of Pediatrics, University of Texas Medical School at Houston, Houston, Texas, United States of America; 2 Shriners Hospital for Children, Houston, Texas, United States of America; University of Florida, United States of America

## Abstract

Mutations in cartilage oligomeric matrix protein (COMP), a large extracellular glycoprotein expressed in musculoskeletal tissues, cause two skeletal dysplasias, pseudoachondroplasia and multiple epiphyseal dysplasia. These mutations lead to massive intracellular retention of COMP, chondrocyte death and loss of growth plate chondrocytes that are necessary for linear growth. In contrast, COMP null mice have only minor growth plate abnormalities, normal growth and longevity. This suggests that reducing mutant and wild-type COMP expression in chondrocytes may prevent the toxic cellular phenotype causing the skeletal dysplasias. We tested this hypothesis using RNA interference to reduce steady state levels of COMP mRNA. A panel of shRNAs directed against COMP was tested. One shRNA (3B) reduced endogenous and recombinant COMP mRNA dramatically, regardless of expression levels. The activity of the shRNA against COMP mRNA was maintained for up to 10 weeks. We also demonstrate that this treatment reduced ER stress. Moreover, we show that reducing steady state levels of COMP mRNA alleviates intracellular retention of other extracellular matrix proteins associated with the pseudoachondroplasia cellular pathology. These findings are a proof of principle and the foundation for the development of a therapeutic intervention based on reduction of COMP expression.

## Introduction

Cartilage oligomeric matrix protein (COMP), also known as thrombospondin 5 (TSP-5), is a low abundance glycoprotein that is found in the extracellular matrix (ECM) of cartilage, tendon, ligament and smooth muscle [1], [2], [3]. Serum and synovial levels of COMP are now used to assess cartilage erosion in osteoarthritis and joint injury [4], [5]. Interest in this extracellular matrix protein increased when it was recognized that mutations in COMP caused pseudoachondroplasia (PSACH) and multiple epiphyseal dysplasia (MED/EDM1) [6], [7]. It had long been recognized that both PSACH and MED chondrocytes retained lamellar appearing material in large rough endoplasmic reticulum (rER) cisternae [8]. This material was subsequently shown to be composed of COMP and other ECM proteins including types II and IX collagens and matrilin-3 (MATN3) [9], [10]. Recently, using flouresence deconvolution analysis, these retained proteins were shown to be organized into a matrix network suggesting that the stalled mutant COMP inappropriately interacts intracellularly with matrix protein partners [9], [10], [11]. The massive retention of intracellular matrix is toxic to the chondrocytes and prolonged rER stress induces apoptosis causing chondrocyte death [12]. The resulting loss of chondrocytes in the growth plate translates into decreased long bone growth and the disproportionate short stature found in PSACH. Intracellular retention and death of chondrocytes causes the loss of these proteins in the ECM [13], [14]. The resulting downstream effect is a disorganized type II collagen network most likely due to the absence of type IX collagen which is needed to crosslink type II collagen [15]. Altogether, the loss of these proteins from the matrix and the poorly organized ECM structure of the articular cartilage contribute to joint abnormalities and early onset osteoarthritis characteristic of both PSACH and MED/EDM1 [14], [16].

In contrast, COMP knock-out mice are normal in all growth parameters with only mild growth plate disturbances and flattening of the articular cartilage surface only found with exercise [16]. This suggests that the presence of mutant COMP has a greater negative impact on skeletal growth and development than the absence of normal COMP. Moreover, these findings also suggest that surpressing intracellular retention of mutant COMP by reducing COMP expression could be used as a therapeutic approach. RNA interference (RNAi) technology is one method to selectively reduce expression of a particular gene.

The challenges to using RNAi therapy in a clinical setting are significant and this barrier is particularly difficult with avascular tissues such as cartilage. Preventing or reducing PSACH cellular pathology will require long-term reduction of COMP expression because chondrocytes produce COMP throughout life [2], [17], [18]. However, there is active research to develop technologies capable of delivering therapeutics including shRNA delivery vectors to cartilage tissue. One system relies upon cell-specific antibodies to attach to viral envelope proteins for specific delivery or nanoparticles to deliver plasmids or siRNAs [19], [20], [21]. Similarly, polypeptide ligands that bind to cartilage are designed for delivery into this difficult tissue (US Patent 7592009). Additionally, topical administration of siRNAs have been shown to deliver sufficient levels of antisense RNAs to reduce the target mRNA in mice joints. Osteopontin siRNA cream prevents severe and irreversible damage to bone and cartilage caused by collagen antibody-induced rheumatoid arthritis in a mouse model [22]. This topical delivery method has not been verified in larger animals where thicker tissue may inhibit sufficient penetration. If this type of therapy is effective in larger animals then it could be used for extended periods due to the non-invasive patient-friendly characteristics of topical applications.

In this study, recombinant lentiviruses encoding the elements necessary for expression of a COMP-specific shRNA were used to provide a continuous supply of shRNA for COMP-specific siRNA duplexes [23]. Three cell types, COS-7, primary human chondrocytes and rat chondrosarcoma (RCS) cells, were used to assay the effectiveness of various COMP-directed shRNAs. COS-7 cells were used to screen and assess the ability of individual shRNAs to reduce COMP expression. Primary human chondrocytes were used to demonstrate the effectiveness of shRNA against endogenously expressed COMP. RCS cells were used to assess the effect of shRNA targeted against human COMP in the presence of other ECM proteins. The results presented here indicate that shRNA directed at COMP reduces expression levels and the cellular phenotypes of rER retention, indicating potential therapeutic utility.

## Results

### Identification of shRNAs that reduce COMP expression

**Figure 1:**
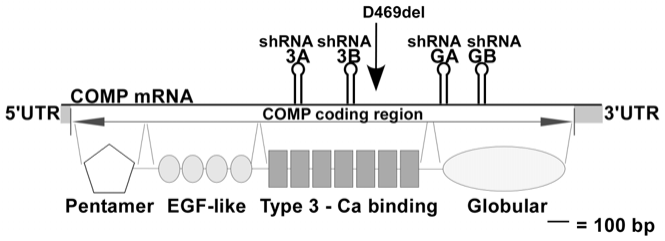
Schematic of human COMP showing shRNA target locations relative to mRNA structure and protein domains. Solid gray blocks are used to indicate the 5′UTR, coding region and 3′UTR of COMP mRNA. The positions of COMP protein domains including the pentamer, epithelial growth factor (EGF)-like, type 3 calcium repeat and globular domains are shown below the protein structure. The position of the common D469del mutation in exon 17B is designated by arrow. Stem-loop hairpins indicate target positions of the shRNAs.

**Figure 2:**
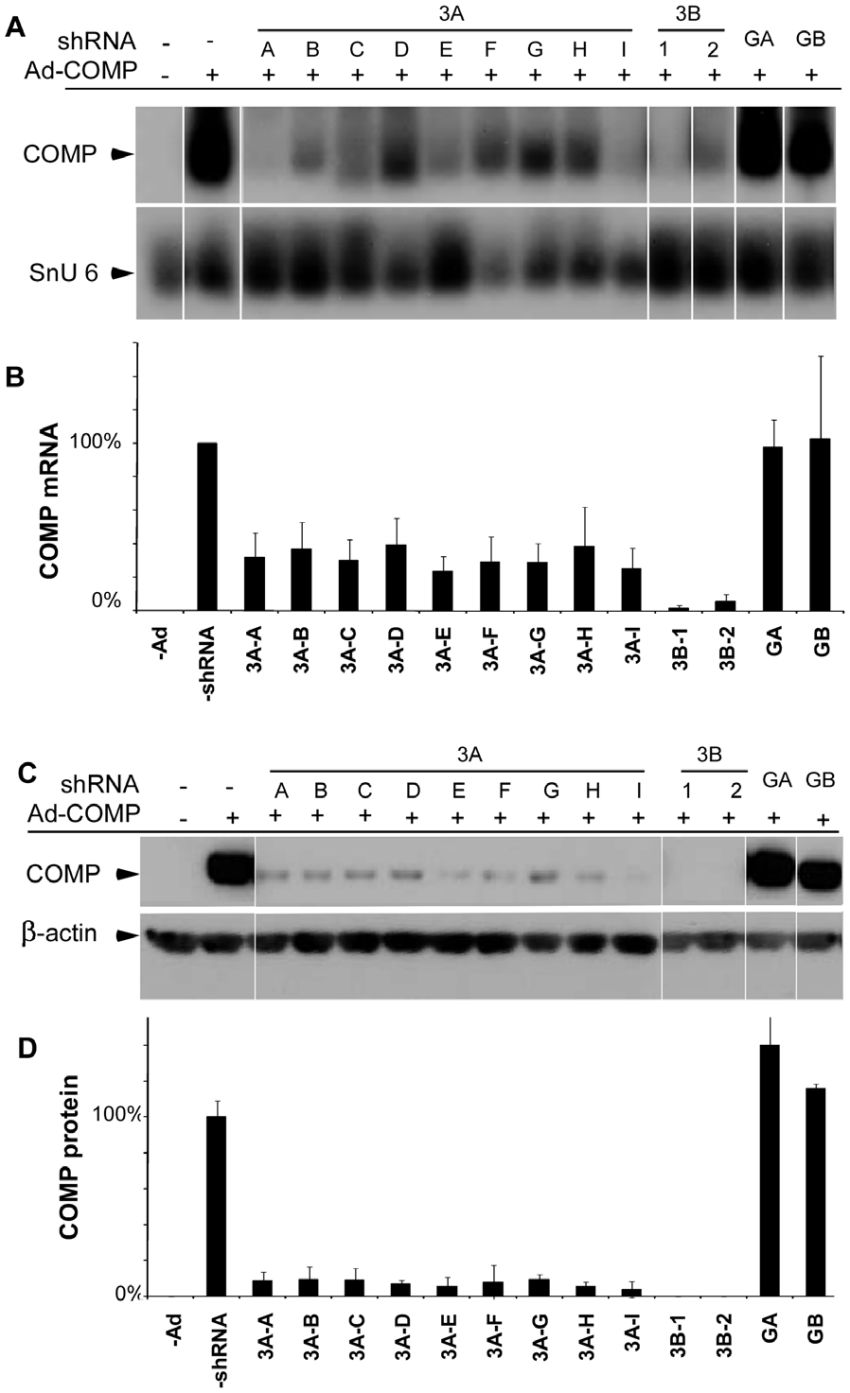
shRNAs directed against human COMP effectively reduce steady-state levels of COMP mRNA and protein. COS-7 cells were stably transfected with shRNAs directed against COMP, and individual clones identified and selected as described in Methods section. Cells were infected with an adenovirus expressing MT-COMP and collected 48 hours after infection. RNA and protein were purified for subsequent analysis. (**A**) Northern blot analysis of steady-state COMP mRNA in transfected cells. (**B**) Quantification of mRNA in a. Absence of a shRNA was set to 100% and all other lanes were compared to the no shRNA reference sample. snU6 mRNA was used to normalize protein loading. (**C**) Western blot analysis. (**D**) Quantification of protein in c in which the level of COMP protein from COS-7 cells expressing recombinant COMP protein in the absence of a shRNA was set to 100% and all other protein levels were compared to this reference. β-actin was used to control for protein loading. All experiments were replicated at least three times.

A panel of four shRNAs with predicted homology to different regions of the human COMP mRNA was screened for the ability to reduce COMP expression. Stable integrants of each U6-shRNA expression cassette in COS-7 cells shown in **Fig. 1** were isolated and cultured as described in Material and Methods section. COS-7 cells with different shRNA integrants were infected with either wild-type (WT)- or mutant (MT)-COMP (D469del common mutation) adenovirus and assessed for COMP knockdown. As shown in **Fig. 2**, COMP mRNA (**A and B**) and protein (**C and D**) were markedly reduced by two shRNAs, 3A and 3B, that target different COMP type 3 calcium-repeat domain sequences (**Fig. 1**). Two shRNA integrants of 3B (3B-1 and 3B-2) reduced COMP mRNA and protein levels to less than 10% of the control compared to integrants of shRNA 3A (3A A–I) that only reduced COMP to approximately 30% of the control. In contrast, the integrants containing GA and GB shRNAs that target globular domain sequences did not reduce steady state WT- or MT-COMP mRNA or protein levels (**Fig. 2** and data not shown). These results demonstrate that targeting the type 3 repeat domain was more effective at reducing COMP than targeting the globular domain.

### shRNA 3B reduces ER stress

**Figure 3:**
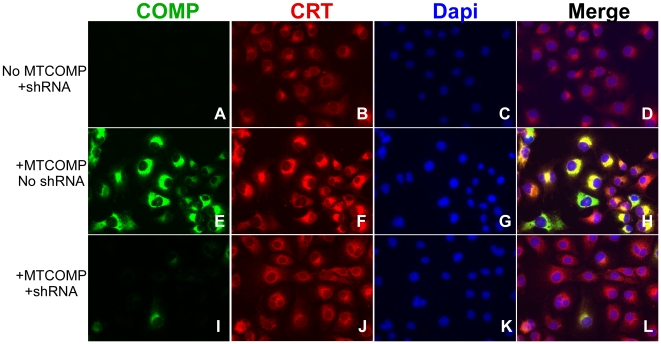
Cells expressing MT-COMP with a COMP-directed shRNA 3B show reduction of intracellular COMP and CRT. COS-7 cells with (**I–L**) and without (**E–H**) shRNA 3B were infected with adenoviruses expressing MT-COMP (**E–L**). Uninfected COS-7 cells with shRNA 3B serves as controls (**A–D**). Cells were grown on coverslips for 48 hours after adenoviral infection and immunostained with COMP and CRT antibodies as described in the Methods section. DAPI (blue) staining was used to localize all of the cells in the field. COMP (green in E) co-localizes (merge-yellow in H) with CRT (red in F).

Previously, we had shown that calreticulin (CRT), a resident rER protein, was associated with retained mutant COMP in the rER of PSACH growth plate chondrocytes [24]. In these experiments, we sought to determine if reduction of COMP mRNA by shRNA would reduce ER stress by assessing CRT as a measure of ER stress in COS-7 cells. Expression of MT-COMP in the absence of shRNA 3B elevates CRT levels, indicating increased ER stress (**Fig. 3F**). Additionally, CRT and MT-COMP co-localize to the ER, demonstrating intracellular retention (**Fig. 3H**). Alternatively, increased ER stress (CRT levels) and co-localization of CRT and COMP were greatly diminished in cells overexpressing MT-COMP in the presence of shRNA 3B (**Fig. 3I–L**). As expected, shRNA 3B was effective at knocking down both WT- and MT-COMP because it was not specifically designed against the mutant COMP mRNA (**Fig. 3** and data not shown). These results suggest that reducing intracellular COMP via knock down of COMP mRNA lessens ER stress.

### shRNAs maintain long-term knock down in the presence of high levels of COMP expression

**Figure 4:**
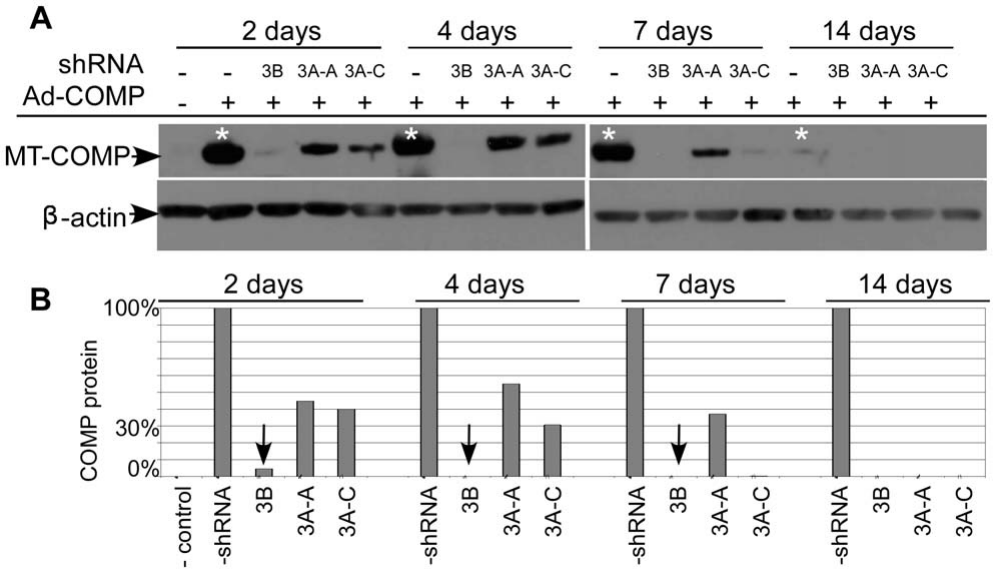
Long-term activity of shRNA COMP knockdown was maintained over 2 weeks. COS-7 cells with shRNA 3B, 3A-A or 3A–C integrant were infected with an adenovirus expressing MT-COMP and protein lysate was collected at 2, 4, 7 and 14 days. (**A**) Western analysis of protein lysates of MT-COMP expression levels at 2, 4 and 7 days. After 14 days, appreciable amounts of COMP were not being produced (*). (**B**) Quantification of COMP protein levels from COS-7 cells expressing recombinant COMP in the absence of a shRNA was set to 100% (*a) and all other lanes were compared to this reference. Levels of β-actin were used to normalize protein loading.

Rescue of the PSACH cellular pathology will require long-term knock down because chondrocytes produce COMP during growth, development and adulthood [2], [17], [18], [25], [26], [27], [28]. In these experiments, the ability of shRNA 3A and 3B to knock down COMP over extended culture time was assessed. COS-7 cells with and without an shRNA integrant were infected with MT- or WT-COMP adenovirus, cell lysates were collected 2, 4, 7, and 14 days post-infection and assayed by Western analysis. The experiment was terminated after two weeks because COS-7 replication and viral degradation diluted the adenoviral COMP expression to very low levels. By 2 days shRNA 3B reduced COMP protein levels by 90%, and COMP expression was not detectable after 4 days of culture (**Fig. 4** *) (only MT-COMP results are shown in **Fig. 4** because WT-COMP knock down was identical). shRNA 3A was approximately three times less efficient than 3B at reducing COMP protein levels. However, this shRNA retained the ability to reduce COMP expression over time (**Fig. 4 A and B**).

**Figure 5:**
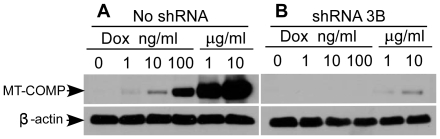
shRNA knockdown was maintained in the presence of high levels of COMP. COS-7 cells with and without 3B shRNA integrant were infected with a DOX-inducible MT-COMP adenovirus. DOX dosage ranging from 1 ng/ml to 10 µg/ml was used. Protein lysate was collected 48 hours after adenovirus infection. (**A**) Western blot analysis of COMP expression in the absence of shRNA 3B. (**B**) Western blot analysis of COMP expression in the presence of shRNA 3B.

We next asked if shRNA 3B would be effective against high levels of COMP expression. In this set of experiments, we used the tetracycline induced MT-COMP adenovirus and varied the dose of doxycycline (DOX) to attain high levels of COMP expression. As can been seen in **Fig. 5A**, MT-COMP was induced in a dose dependent manner and high levels were attained at 1 and 10 µg/ml of DOX. In the presence of shRNA 3B, MT-COMP protein levels were dramatically reduced (**Fig. 5B**). Similar results were found for WT-COMP (data not shown). All together we show RNAi was effective at reducing high levels of COMP and remains effective over time.

### shRNAs efficiently reduce endogenous COMP expression in human growth plate chondrocytes

**Figure 6:**
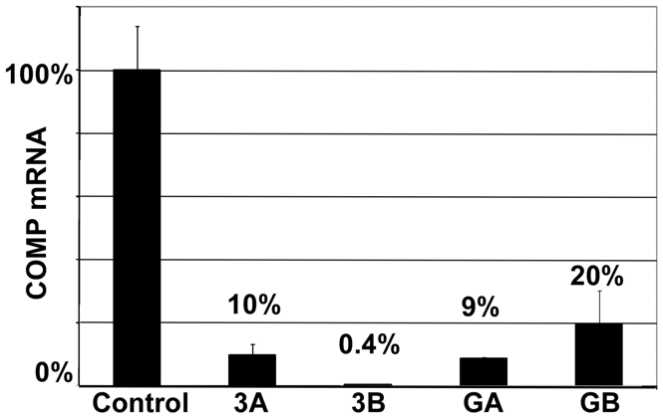
shRNAs directed against human COMP reduces steady-state levels of endogenous COMP mRNA from primary chondrocytes. A population of integrants for each shRNA was isolated using puromycin selection as described in the Methods section. Primary human chondrocytes, with and without an shRNA, were cultured in monolayer for one week and RNA was collected. COMP is expressed in primary human chondrocytes grown in monolayer culture. qRT-PCR was used to quantify the COMP mRNA levels and β-actin mRNA levels were used to normalize the RNA.

We next tested the efficacy of shRNAs to reduce endogenous COMP mRNA levels in human chondrocytes. Chondrocytes containing shRNA integrants directed against COMP were grown in monolayer for one week. As shown in shown in **Fig. 6**, all four of the shRNAs reduced endogenous human COMP mRNA levels by at least 80%. Consistent with our recombinant COMP expression results, shRNA 3B was the most effective at reducing endogenous COMP mRNA levels, a 99% reduction. COMP is expressed at low levels when human primary chondrocytes are cultured in monolayer. In this context, all of the shRNAs directed at COMP work well; however, when COMP expression levels are higher, shRNA 3B out performs the other shRNAs. This result shows that shRNA 3B was equally effective at reducing a wide range of COMP mRNA expression levels.

### shRNA 3B is effective in reducing intracellular retention of MT-COMP and other ECM proteins

**Figure 7:**
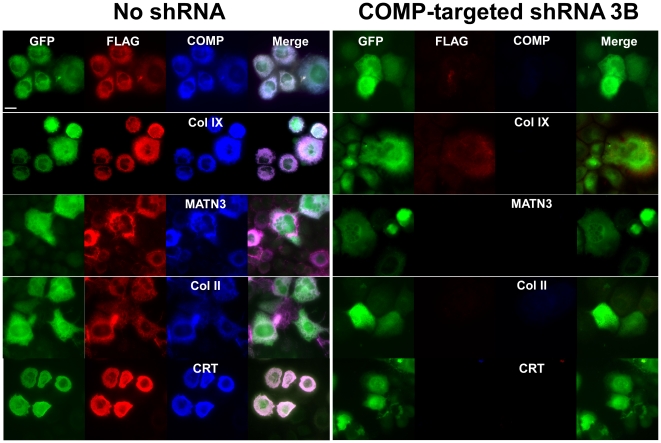
Immunohistochemistry analysis of RCS cells expressing MT-COMP with and without a COMP-directed shRNA 3B. RCS cells with (right panel) and without (left panel) shRNA 3B, grown on coverslips, were infected with adenovirus expressing MT-COMP-FLAG and GFP. Cells were fixed 4 days after adenoviral infection and immunostained with COMP, type IX collagen, MATN3 and type II collagen antibodies as described in the Methods section. GFP was used to identify infected cells. FLAG was used as a marker for recombinant COMP (red) and the rER was delineated with a CRT antibody (blue).

We have previously shown that MT-COMP expression induces the intracellular retention of ECM proteins and formation of intracellular matrix [9], [10]. The goal of these studies was to determine if reduction in MT-COMP synthesis by the most effective shRNA (3B) can prevent and/or eliminate intracellular retention of COMP and other ECM proteins. RCS cells were used in this set of experiments because unlike primary chondrocytes in monolayer that become fibroblastic and do not synthesize matrix proteins, RCS cells maintain a chondrocyte phenotype synthesizing COMP and other ECM proteins [29]. RCS cells with and without shRNA 3B were infected with adenovirus expressing FLAG tagged MT-COMP with and green fluorescent protein (GFP) and fixed after 4 days in culture. DAPI was used to define the total number of cells and GFP was used to identify infected cells. Only infected cells were considered in this analysis because the shRNA targets the human COMP transcript and does not cross-react with rat COMP. As shown in **Fig. 7** (**left panel**), in the presence of MT-COMP, MATN3, types IX and II collagens were co-retained. In comparison, shRNA 3B reduction of MT-COMP also reduced the retention of other ECM proteins retained (**Fig. 7** **right panel**). This result demonstrates that reduction of intracellular MT-COMP by RNAi normalizes the export of the other matrix proteins preventing the premature protein interactions and intracellular matrix assembly. Moreover, expression of CRT was also reduced in cells expressing shRNA 3B. These findings are consistent with the COS-7 results shown in **Fig. 3**.

## Discussion

PSACH is a severe dwarfing condition caused by mutations in COMP that specifically affects growth plate chondrocytes. These mutations interfere with protein folding which initiates the ER stress response causing retention of COMP and other ECM proteins that assemble prematurely into ordered intracellular matrix [9], [10], [11], [13], [14]. The massive intracellular retention of this matrix network causes chondrocyte death and results in diminished limb growth and joint abnormalities. The objective of this study was to determine whether suppression/reduction of COMP expression could resolve the cellular chondrocyte pathology. We show that COMP mRNA and protein levels can be significantly decreased by shRNAs directed against the COMP type 3 repeat domain. Specifically, shRNA 3B was effective at reducing endogenous and recombinant COMP expression and maintaining this reduction for up to ten weeks (data not shown). The shRNA-mediated reduction of MT-COMP expression prevented the development of the PSACH cellular phenotype as demonstrated by the absence of retained COMP and other ECM proteins. This is the first study to show that the PSACH cellular phenotype can be mitigated by RNAi reduction of COMP expression.

Most of the mutations that cause PSACH are in the calcium-binding domain of COMP [6], [30], [31], [32], [33], [34]. These mutations cause increased ER stress, reduced calcium binding, interfere with calcium-dependant protein folding and hamper protein trafficking *in vitro* [13], [18], [24], [35], [36]. COMP is a pentameric protein comprised of five identical monomers assembled in the rER, modified in the Golgi and then exported to the matrix where it is incorporated into the extracellular matrix of the growth plate [1]. COMP mutations have a dominant negative effect because perturbation of the three dimensional structure of the protein activates the unfolded protein response (UPR) [11], [18], [35]. COMP and other ECM proteins accumulate in the ER associated with numerous chaperone proteins, including CRT, protein disulfide isomerase, Grp94 and BiP [24]. Deconvolution analysis showed that the protein retained in the rER of PSACH chondrocytes is organized into an ordered intracellular matrix and this may hinder the ER clearance mechanisms [9], [10]. Prolonged stress due to protein accumulation in the rER ultimately causes premature chondrocyte cell death [11], [13], [37], [38]. Surprisingly, mice lacking COMP have only minor skeletal anomalies, suggesting that absence of COMP is not detrimental to morphogenesis and skeletal development [16]. It is the disparity between the human PSACH phenotype and COMP null mouse phenotype that led us to investigate RNAi as a potential therapy for COMP-related skeletal dysplasias by eliminating all COMP mRNA.

RNAi therapies are being tested in a variety of conditions including, cancer, macular degeneration, movement disorders, heart failure, storage diseases, prion disease, viral infection, chronic pain and addictive-induced behavior [5], [39], [40], [41], [42], [43], [44], [45], [46], [47], [48], [49], [50], [51], [52]. Vascular endothelial growth factor (VEGF)-targeted siRNA therapies for age related wet type of macular degeneration are the closest to clinical application and have progressed to safety and efficacy clinical trials [53], [54]. Age-related wet type of macular degeneration occurs when excess blood vessels behind the retina interfere with vision. Blood vessel growth suppression by silencing of VEGF expression has been shown to improve vision [42], For Gaucher disease, a lysosomal storage disorder, RNAi substrate reduction therapy also shows promise [48]. The therapeutic approach taken in Gaucher cells is to diminish the amount of substrate processed through the lysosome so that the remaining substrate can be cleared by the residual lysosomal enzyme activity [48]. Using a similar approach, we postulated that COMP reduction therapy would decrease the load of improperly folded COMP in the rER, thereby preventing intracellular protein accumulation and subsequent ER stress.

The PSACH and MED/EDM1 cellular pathology occurs for the first 15–18 years which coincides with human skeletal growth [2], [17], [18]. Therefore, long-term knock down of COMP is necessary to suppress PSACH and MED/EDM1 cellular phenotype. In non-dividing cells, exogenously introduced siRNAs are degraded within a few weeks and in dividing cells, siRNAs are diluted to non-effective concentrations within 3–7 days after transfection [54]. In contrast, shRNAs have been shown to reduce target mRNA for longer periods (up to 51 days) [55], [56]. In these studies, using the COS-7 culture system, long-term decreases in COMP expression were sustainable for up to two weeks in the presence of recombinant COMP (**Fig. 4**). In human chondrocytes with shRNA integrants, COMP suppression was maintained up to ten weeks (data not shown). These results suggest that long-term suppression is possible using shRNAs directed against COMP.

COMP is expressed at high levels during skeletal development and long bone growth and therefore it is important that RNAi therapy be capable of reducing high expression levels of COMP [2], [17], [18]. Using a DOX-inducible system, we tested shRNA 3B against varying levels of COMP expression. Knock down was observed with all levels of COMP expression suggesting that shRNA 3B was effective even when target mRNA levels are high and therapeutic intervention would be most needed (**Fig. 5**).

We also demonstrated in RCS cells that shRNA 3B efficiently reduced intracellular retention of MT-COMP, MATN3 and type IX collagen, thereby preventing the development of intracellular matrix that defines the PSACH and MED/EDM1 cellular phenotype. Moreover, in both COS-7 and RCS cells, shRNA 3B reduced the elevated CRT levels that result from the overexpression of COMP. Taken together, these results suggest that reducing mutant COMP mRNA levels may dampen the ER stress and matrix formation in the rER that is potentiated by the presence of mutant COMP.

Overall, we show that suppression of COMP expression by RNAi *in vitro* would be an effective treatment modality for the PSACH and MED/EDM1 chondrocyte pathology. This work provides a proof of principle for the potential development of treatment for this ER storage disorder. Delivery of RNAi therapy to avascular tissues, such as cartilage may be a challenge. In one approach, the delivery of siRNA molecules to bone metastatic tumors was mediated by their attachment to bovine pepsin treated type I collagen (atelocollagen) [57], [58]. This complex was infused and taken up by the tumor. Future delivery mechanisms that target cartilaginous tissue and/or chondrocytes need to be developed. The long-term goal for a treatment modality for PSACH and MED/EDM1 is to prevent premature chondrocyte loss and restore chondrocyte function, which is necessary for normal bone growth and ECM integrity.

## Materials and Methods

### shRNA expression cassettes and recombinant shRNA lentivirus

shRNA lentiviral particles that target human COMP were purchased from the MISSION® TRC-Hs 1.0 (Human) (Sigma, St. Louis, Missouri). MISSION shRNA clones were designed and developed by The RNAi Consortium (TRC) at the Broad Institute of MIT and Harvard University. Each shRNA is a hairpin with 21 base pair long stem and a 6 nucleotide loop. Expression of the shRNA is driven by the U6 promoter. The target region of each shRNA is shown in **Figure 1**. Target sequences are: 3A CCCAGAAGAACGACGACCAAA, 3B ATGCTTGTGACAGCGATCAAC, GA TGTGGGTTACACTGCCTTCAA and GB CAAACGTATTGGCAGGCGAAC.

### Design and generation of a GFP reporter COMP adenovirus

The bicistronic constructs were designed to express MT- or WT-COMP with a FLAG tag moiety (DYKDDDDK) at the carboxy terminus and GFP reporter gene under the control of the cytomegalovirus (CMV) promoter. The D469del mutation, used in this study, is the most common PSACH mutation accounting for approximately 30% of cases [34]. These constructs and viruses were previously used in our PSACH cell culture model system [9]. Using the AdEasy vector system kit (Qbiogene, Irvine, CA) and following manufacturer's guidelines, the expression cassettes were then placed into a replication-defective recombinant adenovirus vector. All constructs were verified by sequencing.

### Design and generation of an inducible recombinant COMP adenovirus

Inducible WT- and MT-COMP expression cassettes were designed so that the level of COMP expression could be modulated by altering DOX concentration in the culture medium. Two subcloning steps were performed to generate the final vector pShuttle-TRE-COMP-CMV-rtTA. First, the CMV-rtTA cassette from pTet-On (Clontech, Mountain View, CA) was inserted into the pShuttle (Stratagene, La Jolla, CA) using PCR (5′CCAGTCACGTAGCGATAGCGGAGTG3′ For and 5′ATAGATCTAAGCTTGGTCGAGCTGATACTTCCCG 3′ Rev) and digested with *Xho* I and *Bgl* II. The resulting fragment was ligated into complementary sites in pShuttle (*Sal* I and *Bgl* II) (Stratagene, La Jolla, CA). Next, the WT- or MT-COMP-FLAG gene driven by the tetracycline responsive element (TRE) was inserted into pShuttle-CMV-rtTA to produce the final construct pShuttle-TRE-COMP-CMV-rtTA. All constructs were verified by sequencing. The AdEasy vector system kit (Qbiogene, Irvine, CA) was again used as described above. Recombinant adenovirus were subsequently produced, isolated and quantified.

### Cell culture techniques

COS-7 (Sigma, St. Louis, Missouri) and RCS [59] cells were cultured in DMEM with 10% FBS and antibiotics and passaged until 80% confluent using standard methods. Primary human costochondral chondrocytes were collected from patients undergoing pectus excavatum surgery and obtained from the Iowa Skeletal Dysplasia Cell Bank with IRB approval. These cells were cultured using standard conditions in DMEM with 20% v/v FBS as previously described [36].

### Generation of cells expressing shRNA

Lentivirus particles containing a shRNA were used to infect RCS and COS-7 cells at multiplicity of infection (MOI) of 0.1 to insure that single colonies of integrants could be isolated. Cells were cultured for three days in non-selective media and puromycin was then added to select stable integrants. Single colonies were moved to separate dishes and grown in standard culture media with puromycin for these experiments. Primary human costochondral chondrocytes were infected at MOI of 1 with shRNA lentiviruses and grown for five days in non-selective media and then in puromycin to select cell populations that contained an shRNA. All human primary chondrocytes in these experiments are from a population of shRNA integrants.

### Transfection of cells with recombinant adenoviruses

COS-7 cells with and without an shRNA integrant were cultured until 70–80% confluent and infected with either the WT- or MT-COMP-GFP or DOX inducible WT- or MT-COMP-FLAG adenovirus (MOI 15 for all adenovirus infections) in DMEM with 2% v/v FBS. Cells were harvested 48 hrs later or at time points required for the experiments. RCS cells with and without a shRNA integrant were plated on to coverslips and 24 hrs later treated with 1 µg/ml hyaluronidase for 1 hour at 37°C and then infected with MT-COMP-FLAG (GFP) adenovirus (MOI 400–800) in OPTI-MEM media (Gibco, Carlsbad, CA).

### mRNA and protein analyses

Northern and Western analyses were used to assess mRNA and proteins in all experiments using standard techniques. Sample loading in Northern and Western analyses was normalized using Snu-6 and β-actin, respectively. Westerns were performed using anti-COMP rabbit polyclonal antibody (Kamiya, Thousand Oaks, CA) and detected using ECL Plus (GE Healthcare Biosciences, Pittsburgh, PA).

### Histology and immunohistochemistry

RCS cells and COS-7 cells were grown on coverslips and fixed using 4% **v/v** paraformaldehyde after 4 days and 100% methanol after 2 days, respectively, at 4°C for 20 min. All coverslips were incubated overnight at 4°C with the primary antibodies (1∶200). Table 1 lists the antibodies used in this study. Primary antibodies were detected using fluorochrome conjugated secondary antibodies (1∶400) (Table 1). Co-immunostaining and imaging was performed as previously described and all exposure times are equal [9], [10]. Coverslips were mounted using ProLong Gold anti-fade reagent (Molecular Probes, Carlsbad, CA) or DAPI (4′-6-Diamidino-2-phenylindole) prolong gold anti-fade reagent staining to detect nuclei.

**Table 1:** Antibodies used in studies.

Cell type	Primary Antibody	Secondary Antibody
**Fig. 3** ** COS-7**	COMP rabbit polyclonal (K)	Anti-rabbit alexa flour 488 (MP)
COS-7	CRT goat polyclonal (SC)	Anti-goat alexa flour 594 (MP)
COS-7	FLAG mouse monoclonal (S)	Anti-mouse alexa flour 594 (MP)
**Fig. 7** ** RCS**	COMP rabbit polyclonal (K)	Anti-rabbit Marina Blue M10992 (MP)
RCS	Col IX rabbit monoclonal (C)	Anti-rabbit alexa flour 647 (MP)
RCS	MATN3 goat polyclonal (R&D)	Anti-goat alexa flour 647 (MP)
RCS	FLAG Goat polyclonal (Ab)	Anti-goat alexa flour 594 (MP)
RCS	Col II mouse monoclonal (NM)	Anti-mouse alexa flour 647 (MP)
RCS	CRT goat polyclonal (SC)	Anti-goat alexa flour 647 (MP)

Ab = Abcam, Cambridge, MA, C = Calbiochem, San Diego, CA, K = Kamiya, Thousand Oaks, CA, MP = Molecular Probes, Carlsbad, CA, NM = NeoMarkers, SC = Santa Cruz, Santa Cruz, CA, and R&D = R&D Systems, Minneapolis, MN.

### Quantitative real-time RT-PCR

RNA from chondrocytes with and without an shRNA integrant was collected for RT-PCR assays after one week in culture. Quantitative real-time RT-PCR (qRT-PCR) was performed utilizing the 7700 Sequence Detector (Applied Biosystems, Foster City, CA). DNA contamination was removed using amplification grade DNAse (Invitrogen, Carlsbad, CA) following manufacturer's instructions. Each assay was replicated three times and each sample was measured in triplicate including a control without reverse transcriptase. The final data were normalized to β-actin ×100 (percent of the normalizer transcript).
